# Processes of Removing Zinc from Water using Zero-Valent Iron

**DOI:** 10.1007/s11270-015-2617-x

**Published:** 2015-10-05

**Authors:** Tomasz Suponik, Antoni Winiarski, Jacek Szade

**Affiliations:** Silesian University of Technology, Akademicka 2, 44-100 Gliwice, Poland; University of Silesia, Uniwersytecka 4, 40-007 Katowice, Poland

**Keywords:** Zero-valent iron, Zinc, X-ray photoelectron spectroscopy, Diffraction, Surface charge

## Abstract

Zero-valent iron has received considerable attention for its potential application in the removal of heavy metals from water. This paper considers the possibility of removal of zinc ions from water by causing precipitates to form on the surface of iron. The chemical states and the atomic concentrations of solids which have formed on the surface of zero-valent iron as well as the type of the deposited polycrystalline substances have been analyzed with the use of X-ray photoelectron spectroscopy (XPS) and X-ray diffraction (XRD), respectively. The BET surface area, the pH at point of zero charge (pH_PZC_), the ORP of the solutions, and the pH and chemical concentrations in the solutions have also been measured. Furthermore, the paper also considers the possibility of release of zinc from the precipitates to demineralised water in changing physicochemical and chemical conditions. In a wide range of pH values, Zn_*x*_Fe_3 − *x*_O_4_ (where *x* ≤ 1) was the main compound resulting from the removal of zinc in ionic form from water. In neutral and alkaline conditions, the adsorption occurred as an additional process.

## Introduction

Zinc is a metal which finds its application in multiple industries. The minerals most commonly exploited for the purposes of zinc production are sphalerite, smithsonite, and calamine. Zinc is essential for human metabolism, but may also pose certain risks. Free zinc ions in solutions are highly toxic to bacteria, plants, invertebrates, and even vertebrate fish (Ronald 1993).

Wastes resulting from the mining of non-ferrous metal ores and hard coal as well as from the manufacturing process of non-ferrous metals accumulated at industrial waste disposal sites contain zinc minerals and, as a result, may affect the quality of ground and surface waters in the southern provinces of Poland. Meanwhile, the Water Framework Directive (Directive 2000/60/EC of the European Parliament and of the Council) issued in October 2000 commits European Union members states to achieve a good qualitative and quantitative status of all water bodies by 2015. Therefore, the problem associated with waste dumps in Silesia, Poland, and their impact on ground and surface waters must be solved as soon as possible. The pH of the groundwater under the waste disposal sites for wastes resulting from the mining of hard coal and non-ferrous metal ores and from the manufacture of non-ferrous metals is slightly acidic and rather neutral or alkaline, respectively.

Permeable reactive barrier technology with the use of zero-valent iron (ZVI) as a reactive material may be applied to remove free zinc ions from groundwater and thus to protect the surface waters. In this technology, the contaminants are removed from the aquifer during the flow of the groundwater through a permeable reactive barrier (PRB) filled with a reactive material. The application of zero-valent iron to remove zinc in cationic form from water has been investigated before (Morrison et al. 2002; Wilkin and McNeil 2003; Kishimoto et al. 2011; Rangsivek and Jekel 2005; Bruzzoniti and Fiore 2014). This research revealed the effectiveness of ZVI in the zinc removal process. The literature, however, includes conflicting reports regarding the mechanism of that process. For example, according to Kishimoto et al. (2011), the mechanism of zinc removal with the use of ZVI powder is as follows: ZVI firstly corrodes and oxidizes into ferric ions by means of dissolved oxygen. The ferric ions then precipitate as iron hydroxide onto the surface of the ZVI powder. Zinc ions adsorb on and/or co-precipitate with the iron hydroxide. The iron hydroxide is then finally oxidized and transformed into iron oxides. Meanwhile, Wilkin and McNeil (2003) and Oh et al. (2007) claim that the rapid removal of heavy metals using zero-valent iron in water contaminated by acid mine drainage proceeds due to the adsorption onto the surface of iron metal or onto iron corrosion products. In the research presented in a paper by Li and Zhang (2007), the predominant removal mechanism for metal ions with a standard potential very close to or more negative than that of iron is sorption/surface complex formation. At this point, it should be made clear that the type of process obviously depends on the chemical and physicochemical composition of the solution. In general, sorption as a removal mechanism is not preferred because soluble zinc Zn(II) remains in its more soluble oxidation state, and in case of a change in the physicochemical conditions, it may be released back to the environment.

In relation to the above, the matters that could raise interest in the view of ZVI application are the types of products formed on the surface of ZVI as a result of zinc removal from water characterized by low, neutral, and high pH values, the possibility of zinc release from the surface of ZVI to demineralised water upon a change in the physicochemical and chemical conditions, as well as the type of the dominant processes that result in the removal of Zn(II) from water. These issues have been discussed in the study.

According to Eh-pH diagrams of the Zn-O-H system presented by Takeno (2005), zinc in solutions exists mainly in its divalent ionic form up to the pH value of 8.5. Between the pH of 8.5 and ca. 11, Zn(OH)_2_ and the cationic species such as Zn(OH)^+^ become increasingly effective. In the pH ranges analyzed in the study, zinc ions were expected to occur in divalent form during the experiments.

## Materials and Method

In order to remove cationic zinc from solutions at various pH values, zero-valent iron constituted the iron samples. The samples used in the batch tests were made of steel (in accordance with EN 10131:2006, “cold rolled uncoated low carbon and high yield strength steel flat products for cold forming”). The samples were square-shaped, made of cold-rolled steel sheet (0.5 mm thickness), and their dimensions were 5 × 5 mm. Just before their use, the samples were immersed in concentrated nitric acid for ca. 120 s and in demineralised water for ca. 120 s, immediately after that they were used in the batch tests. The tests were conducted in a programmable MULTI BIO RS-24 BIOSAN rotator equipped with plastic tubes filled with zinc solution (so as to eliminate the headspace—a gaseous phase above the solutions; the volume of the solution amounted to 58 cm^3^) with the initial pH of 4.5, 7.0, and 9.5 and with iron samples—only one iron sample was introduced to each plastic tube. The pH levels of the solutions were adjusted by slow titration with ultra-pure sulphuric acid solution (0.1 M) or with ultra-pure sodium hydroxide solution (0.1 M). Zinc ion solutions were prepared by adding the desired amounts of metal salt (ZnSO_4_·7H_2_O) into bottles and pouring distilled water. The concentration of zinc in the solutions at pH levels of 4.5, 7.0, and 9.5 amounted to 5.57, 7.06, and 6.12 mg/dm^3^, respectively. The intention of the authors was to exceed the allowable concentration of zinc by several times within the meaning of legal regulations provided in the Journal of Laws (2014, item 1800). This value in the discharged water is 2 mg/dm^3^.

Dissolved oxygen (DO) has a significant impact on the behavior of Zn in the groundwater. The concentration of DO in groundwater (in an unconfined aquifer) located in the vicinity of coal waste disposal sites in the south of Poland amounted to ca. 6 mg/dm^3^ (based on own measurements—the samples of water were taken by the authors using a WHG-168 STALTECHNIKA drilling rig. The aquifers were located at a depth of about 10 m below the surface of the ground). In order to reduce the concentration of oxygen in the samples of water and to adjust their condition to the condition of the contaminated aquifer, the solutions were heated before using them in the batch tests (to the temperature of 308 K). This way, the initial concentration of DO amounted to ca. 6 mg/dm^3^. The ambient air temperature in the laboratory was ca. 295 K.

After filling, the plastic tubes were sealed with corks and the first batch tests were carried out. Each sample had to undergo 5 min of orbital rotation in a programmable rotator (with a speed range of 20 rpm) after which a 6-s reciprocal motion (with a turning angle of 90°) with a vibration motion followed. This sequence of shaking was repeatedly reiterated. The sequence continued for 24 h (after this time, constant values were achieved). After shaking the samples, the solutions were passed through dense filters and assessed. The pH, oxidation-reduction potential (ORP), DO, and conductivity were measured using Knick PORTAMESS meters. The quantitative analyses of chemicals in solutions were carried out for the following:

- Zn_total_, with the use of DR5000 Hach Lange UV-Vis Spectrophotometer—Zincon Method; method 8009 of Hach Co.; test results are measured at 620 nm. The concentration of zinc in the solutions was measured in a spectrophotometer as a total value (Zn_total_). Since there was no zinc speciation (at the beginning) in the solutions other than the Zn(II), the removal of the zinc in the second oxidation state has been assessed in the article.
- Fe(II) with the use of DR5000 Hach Lange UV-Vis Spectrophotometer—1, 10 Phenanthroline Method; method 8146 of Hach Co.; test results are measured at 510 nm.
- Fe_total_ with the use of DR5000 Hach Lange UV-Vis Spectrophotometer—FerroVer Method; method 8008 of Hach Co.; test results measured at 510 nm. The concentration of dissolved Fe(III) was calculated as a difference between Fe_total_ and Fe(II).
- SO_4_^2−^, DR5000 Hach Lange UV-Vis Spectrophotometer—SulfaVer 4 Method; method 8051 of Hach Co.; test results measured at 450 nm.
  The measurements were carried out twice. The results, shown in Table 1, were calculated as an arithmetic mean.

**Table 1:** Physicochemical parameters and concentrations of chemicals in solutions used in the batch tests for the initial pH = 4.5, 7.0, and 9.5

Stage of the batch tests	pH	Cond., uS/cm	ORP, mV	DO, mg/dm^3^	Zn, mg/dm^3^	Fe(II), mg/dm^3^	Fe(III), mg/dm^3^	SO_4_^2−^, mg/dm^3^
Initial pH = 4.5								
Initial values	4.53	137.8	252	6.1	5.57	BDL	BDL	10.2
Values after the first batch tests	5.27	132.4	155	5.0	2.10	0.26	3.22	9.4
Values after the second batch tests	6.83	24.5	124	5.2	0.03	0.15	0.48	1.3
Initial pH = 7.0								
Initial values	7.02	110.1	186	5.8	7.06	BDL	BDL	10.2
Values after the first batch tests	6.31	115.4	128	5.2	1.93	0.10	1.04	9.9
Values after the second batch tests	6.95	17.8	121	5.0	0.04	0.06	0.31	0.6
Initial pH = 9.5								
Initial values	9.48	98.4	146	6.2	6.12	BDL	BDL	9.3
Values after the first batch tests	6.85	104.7	138	5.5	0.17	0.04	0.10	9.1
Values after the second batch tests	7.08	11.6	120	5.2	0.04	0.02	0.08	BDL

*BDL* below detection limit
  After shaking, all the iron samples were pulled out, washed with demineralised water, wiped gently with a paper towels and dried in an exsiccator. In order to identify the products formed on the surface of the samples and to determine their affinity with zinc in ionic form, the following tests were conducted on iron samples before and after the batch tests:
- Determination of crystalline substances present on the surface of iron samples before and after the batch tests. These measurements were carried out with the use of X-ray diffraction (XRD). For the analysis of solid objects, EMPYREAN X-ray Multipurpose Diffractometer by PANalytical was used. The diffractometer was equipped with PreFIX (pre-aligned, fast interchangeable X-ray) modules allowing for an effortless change in the optical path. The PDF4+ database was used for the identification of chemical compounds. Crystal lattice parameters were measured.
- Identification of elements and determination of atomic concentrations of solids located on the surface of iron samples formed before and after the batch tests. These measurements were carried out with the use of X-ray photoelectron spectroscopy (XPS). This method allowed for the identification of the elements (except for H and He) and their chemical states as well as for the calculation of their atomic concentrations. Measurements were performed using a PHI 5700/660 Multipurpose Electron Spectrometer based on two separate test chambers joined by an UHV transfer system by Physical Electronics using a monochromatized Al_Kα_ radiation (hν = 1486.6 eV). The energy resolution of the spectrometer equipped with a hemispherical energy analyzer was approximately 0.3 eV. The anode was operated at 15 kV and 225 W. Survey and multiplex high-resolution spectra (HRES; multiplex) were measured in ultrahigh vacuum. High-resolution spectra were fitted using mixed Gaussian and Lorentzian functions and Shirley background with the application of MultiPak program. The range in survey mode was from −2 to 1400 eV. The measurement parameters for survey mode and HRES (high-resolution mode) were respectively pass energy 187.85 and 23.50, step 0.800 and 0.100 eV, and time per step 20 and 100 ms. The sizes of the analyzed areas were 1.5 × 2.5 mm (to identify the trends in case different pH values of the solutions were used) and the diameter was 0.8 mm (to compare the main precipitates which appeared on the samples after the batch tests). The binding energy of the XPS lines was normalized to the binding energy of C1s = 285 eV. Lines were standardized to be of the same height.
- Determination of the specific surface area of the iron samples before and after the batch tests. The measurements of multi-point BET surface area were carried out with the use of Micromeritics Gemini 2360 Surface Area Analyzer. The method utilized a flowing gas technique in which the gas used in the analysis (nitrogen) was introduced into a tube containing the iron sample and into a balance tube at the same time. The principle of measurement consisted in the adsorption of nitrogen on the surface of the sample at a constant temperature of liquid nitrogen (77–78 K). An identical internal volume and ambient temperature of both tubes was maintained. The only difference was the pressure of the sample in the sample tube. The measurement range of the analyzer: specific surface from 0.01 m^2^/g. In order to remove impurities and moisture, the samples were dried at 383 K before the measurements.
- Determination of the affinity of zinc in ionic form with shells coating the iron sample by measuring the pH at the point of zero charge (pH_PZC_). The point of zero charge was obtained using batch equilibrium method. This parameter describes the condition (pH value) in which the electrical charge on the surface of ZVI is zero. The testing procedure was as follows: iron samples obtained from the batch tests (only from the first batch tests) were immersed in demineralised water for ca. 120 s and then shaken (separately) in plastic tubes for 24 h with the following: (1) 58 cm^3^ of 0.01 M KNO_3_ solution and (2) 58 cm^3^ of 0.01 M KNO_3_ and 10^−5^M Zn(II) solution, at different initial pH values. A suitable amount of Zn(NO_3_)_2_ was added to the solution to achieve a proper concentration of Zn(II). The applied sequence of shaking was similar to the previous ones (performed with a programmable MULTI BIO RS-24 BIOSAN rotator). The initial pH levels of the solutions were adjusted by slow titration with ultra-pure potassium hydroxide solution (0.1 M) or with ultra-pure nitric acid solution (0.1 M) while keeping the ionic strength constant. The amount of H^+^ or OH^−^ ions adsorbed by iron samples was calculated from the difference between the initial and the final concentrations of H^+^ or OH^−^ ions (Babić et al. 1999). The pH was measured with the use of Knick PORTAMESS 913 meter and the SenTix 41 electrode (Suponik 2015b, in press).

In order to assess the capability of metals to be released from the precipitates found on the surface of iron samples to demineralised water and to identify the products formed on the surface of the samples after this process, tests using a programmable rotator were carried out for a second time, in a manner similar to the one described before. The only difference was that the iron samples used for removing metals from solutions (after finishing the first batch tests) were inserted into 58 cm^3^ of demineralised water (DW) poured into plastic tubes. The samples in the second batch tests were shaken in the same way as in the case of the first batch tests. The study conducted after the second test was similar, except for the fact that the pH_pzc_ measurements were omitted.

## Results and Discussion

### Study of Water

As presented in Table 1, the concentrations of zinc in water measured after the first tests decreased in a more rapid manner in case of higher pH levels of solutions. This observation was also made in the papers by Kishimoto et al. (2011), Rangsivek and Jekel (2005), and Suponik and Blanko (2014). What is noteworthy here is the fact that after the second batch tests, the zinc from the iron sample was not released to demineralised water. The compounds formed on iron in the first tests were fixed. They were not dependent on previous pH values. The final pH levels after the second tests were similar in case of all iron samples and amounted approximately to 7. The pH of DW amounted to 6.8. After the first tests, also, this parameter had the tendency to approach the value of 7. The parameter increased for low values and decreased for higher ones.

Kishimoto et al. (2011) studied the desorption of zinc from ZVI using sulphuric acid and citric acid solutions. During the backwashing, they observed rapid desorption of zinc when the citric acid solution was used, whereas no desorption was observed when the sulphuric acid was applied. Their conclusion was that the zinc adsorption layer on ZVI was stable in diluted sulphuric acid and became unstable in a reducing agent such as citric acid. This conclusion is consistent with the results obtained in studies using demineralised water.

As the previous work indicates (Suponik 2015a), the lower were the values of pH in the tests, the higher were the initial values of ORP in the water and the faster was the decrease of this potential after the first batch tests. The ORP of pure DW amounted to 159 mV, while in the case of water after the second batch tests, the value reached 120 mV. A slight decrease in oxygen concentration may also be noticed after both of the batch tests, which indicates that the course of redox reactions involves oxygen. The initial concentration of oxygen in DW amounted to 6.4 mg/dm^3^.

The oxidation of Fe(0) proceeds faster in low pH (Kowal and Świderska-Bróż 1996; Kishimoto et al. 2011), which is evidenced by the fact that more ions of Fe(II) iron appeared after the first batch tests in water characterized by a lower initial value of pH. This occurred mostly due to processes described by reactions 1 and 2. A similar correlation was observed in case of the second batch tests, though the concentrations of Fe(II) were lower. This was probably caused by the thin shells which covered the ZVI.

1 $$ 2F{e}^0+{O}_2+2H{}_2O\to 2F{e}^{2+}+4O{H}^{-} $$

2 $$ {\mathrm{Fe}}^0+2{\mathrm{H}}_2\mathrm{O}\to\ {\mathrm{Fe}}^{2+}+{\mathrm{H}}_2+2{\mathrm{OH}}^{-} $$

In the presence of dissolved oxygen in water, however, Fe(II) is oxidized to Fe(III), which then may produce either FeOOH, Fe(OH)_3_, Fe_2_O_3_, or Fe_3_O_4_ together with Fe(II)—each in solid form.

3 $$ 2{\mathrm{Fe}}^{2+}+\raisebox{1ex}{$1$}\!\left/ \!\raisebox{-1ex}{$2$}\right.{\mathrm{O}}_2+2{\mathrm{H}}^{+}\to 2{\mathrm{Fe}}^{3+}+{\mathrm{H}}_2\mathrm{O} $$

Due to the presence of oxygen in the solutions (Table 1), the concentrations of Fe(III) in almost all samples were several times higher than the concentrations of Fe(II). As it was reported by Kishimoto et al. (2011) and Rangsivek and Jekel (2005), zinc removal by ZVI was enhanced by the presence of dissolved oxygen.

In accordance with the results presented in Table 1, the sulfates in the first batch tests have been removed from the water to a very small extent. Thus, the sulfates were probably not present on the iron sample in the second tests. The release of the sulfates to the water in these tests was very low—especially at higher pH values.

### XRD

Upon visual inspection, the surfaces of the iron samples applied in the first and second tests were covered with gray and yellow-brown precipitates. The amount of yellow-brown deposits seemed to be higher for lower initial pH values of the solutions. In case of the samples immersed in the solutions of pH amounting to 9.5, spots of metallic iron not covered by precipitates were visible. The amount of gray precipitates was similar in all the samples.

**Fig. 1:**
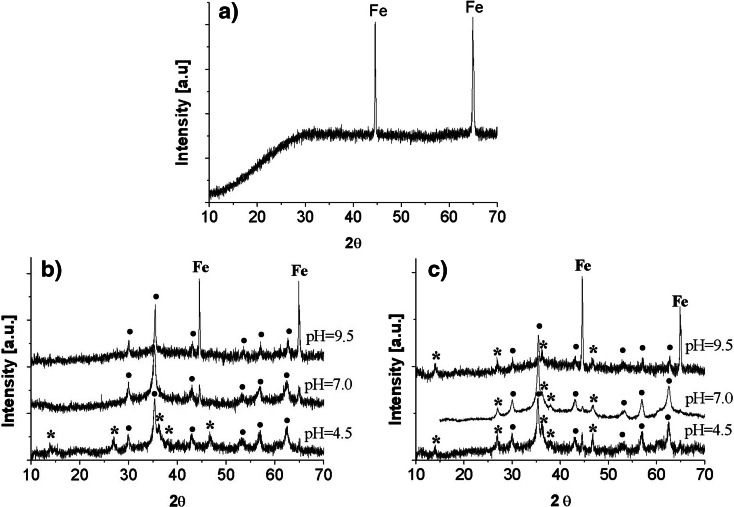
XRD graphs of iron samples: **a** before the batch tests; **b** after the first batch tests for the initial pH of the solution amounting to 4.5, 7.0, and 9.5; and **c** after the second batch tests for the initial pH of the solution amounting to 4.5, 7.0, and 9.5.

Figure 1 presents the diffraction of X-rays on iron samples before and after the first and second batch tests. Based on Fig. 1a, it may be claimed that iron was found on the surface of iron samples assessed before the batch tests (as it was expected). The network parameters of pure iron are similar (very close) to the parameters of many alloys in which iron is the main component.

Pure iron was also present in samples immersed in solutions characterized by higher initial pH levels, especially at pH = 9.5 (in Fig. 1b, c, the theta angle for Fe(0) amounts to 44.6°, 65.0°, and 82.3°). In both the first and the second batch tests for the initial solutions with the pH = 9.5, the samples exhibited many spots not covered by precipitates.

On the contrary, lepidocrocite γ-FeO(OH) was identified mainly in case of lower pH values, especially when the pH of the solutions was 4.5. According to the XRD graphs plotted for the first and second tests, the theta angle in Fig. 1b, c for γ-FeO(OH) amounts to 14.1°, 27.0°, 36.3°, 38.1°, and 46.8°. γ-FeO(OH) may correspond to the yellow-brown precipitates on the samples, occurring mainly in lower pH of the solutions that were used. Lepidocrocite is a common phase often forming as a result of iron corrosion process in the presence of dissolved oxygen (Kamolpornwijit et al. 2004). γ-FeO(OH) was also found on the surface of ZVI after the removal of copper and zinc ions from water (Rangsivek and Jekel 2005). The presence of lepidocrocite indicates that the adsorption of zinc may have occurred as an additional process resulting from the uptake of this metal.

According to the XRD graphs (Fig. 1b, c) obtained after the first and second batch tests, zinc was present in the Zn_*x*_Fe_3 − *x*_O_4_ compound (where *x* ≤ 1). Diffraction lines obtained from the XRD tests correspond to the compounds, which can be expressed as Zn_*x*_Fe_3 − *x*_O_4_, because the diffraction lines of compounds such as Zn^2+^Fe^3+^_2_O_4_, Zn^2+^_0.35_Fe^2+^_0.65_Fe^3+^_2_O_4_, and Zn^2+^_0.4_Fe^2+^_0.6_Fe^3+^_2_O_4_ are very similar. Theta angles for ZnFe_2_O_4_ are 30.0°, 35.4°, 43.0°, 53.2°, 56.8°, and 62.4°. It should be added here that the same pattern was exhibited by all the applied samples, irrespective of the initial pH of the solutions. Zn_*x*_Fe_3 − *x*_O_4_ may correspond to the gray-colored precipitates.

### XPS

**Fig. 2:**
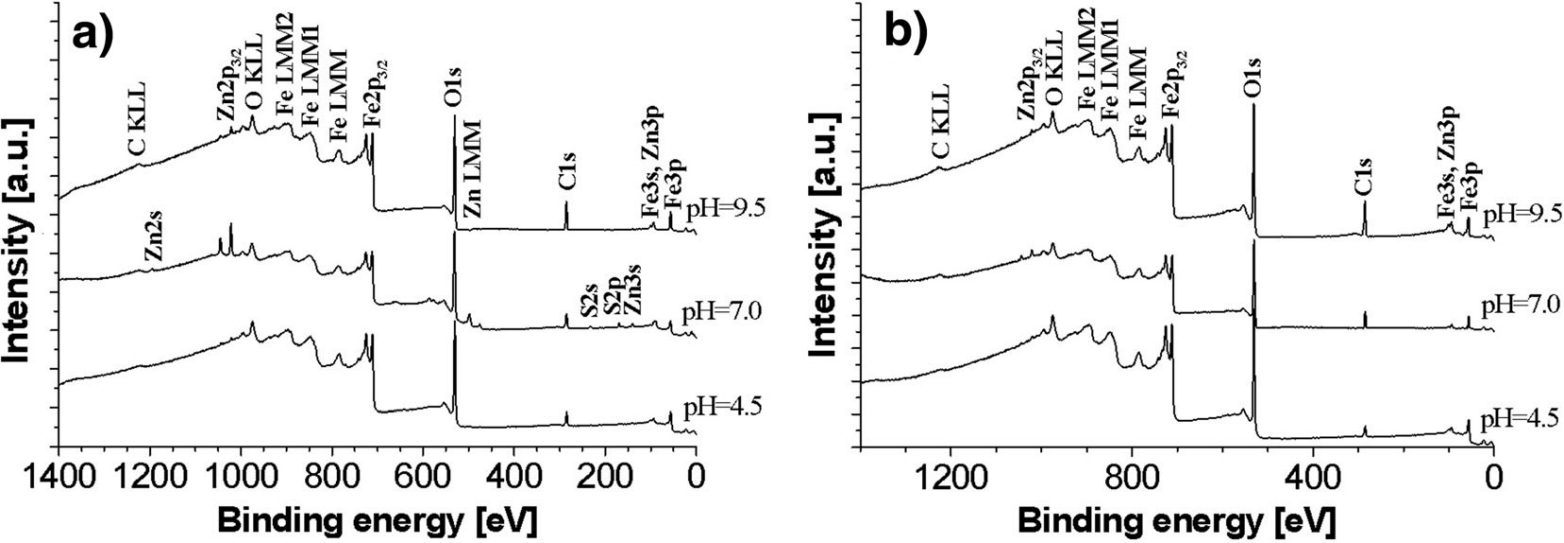
XPS graphs of the iron samples: **a** after the first batch tests for the initial pH of the solution amounting to 4.5, 7.0, and 9.5 and **b** after the second batch tests for the initial pH of the solution amounting to 4.5, 7.0, and 9.5

Table 2 presents the atomic concentrations of the elements on the iron samples before the study and after the first and second batch tests. Each assessed sample contained carbon and oxygen. It is possible that the oxygen was contained in precipitates in the form of metal oxides and metal hydroxides, while carbon and oxygen in compounds in which the following bonds occur (in very small quantities): -C-O- and -C=O (binding energy: C1s = from 286.51 to 288.64 eV, see Fig. 2). The main contribution to the C1s line is constituted by hydrocarbons deposited on the surface. This always occurs when a sample is stored in atmospheric conditions. The binding energy of the C1s line resulting from the presence of hydrocarbons amounts to 285.0 eV.

**Table 2:** Atomic concentrations of elements (expressed as percentages) on the iron samples before the study and after the first and second batch tests for the initial pH of the solutions amounting to 4.5, 7.0, and 9.5—on the basis of XPS measurements

Elements	Type of the study												
	Values before the study, in atom %	Values after the first and second batch tests—for the initial pH = 4.5, in atom %				Values after the first and second batch tests—for the initial pH = 7.0, in atom %				Values after the first and second batch tests—for the initial pH = 9.5, in atom %			
	XPS—survey mode	XPS—survey mode		XPS—HRES, multiplex		XPS—survey mode		XPS—HRES, multiplex		XPS—survey mode		XPS—HRES, multiplex	
		After first test	After second test with DW	After first test	After second test with DW	After first test	After second test with DW	After first test	After second test with DW	After first test	After second test with DW	After first test	After second test with DW
C	62.80	16.09	12.10	16.99	12.41	20.50	20.47	21.25	25.03	27.24	30.46	28.13	27.49
O	29.49	55.35	56.09	54.77	57.06	51.94	54.17	52.08	50.36	48.52	46.48	46.90	48.20
S		0.36	0.07	0.59	0.08	2.45		2.73					
Fe	7.71	27.86	31.63	27.23	30.15	20.16	24.28	19.40	23.59	23.54	22.79	24.13	24.05
Zn		0.35	0.11	0.42	0.30	4.95	1.08	4.54	1.02	0.69	0.26	0.84	0.25

The oxygen present in the compounds on the surface of the iron samples originated from solutions used in the analysis in the first and second batch tests. The presence of carbon and remaining amounts of oxygen has been explained by the contamination of the samples (by atmospheric air and other factors) during their transport for the measurements. For that reason, the atomic concentrations of oxygen and carbon presented in Table 2 have not been taken into consideration in further analysis.

The results of the tests presented in Table 2 have also shown that zinc and iron formed compounds on the surface of all the samples used in the first and second batch tests. Although the assessments concerned a small area of the samples, it can be concluded that the zinc concentration on iron samples was lower in case of samples introduced to the solution with pH = 4.5. This is consistent with the observation that in lower pH levels of solutions, the removal of zinc proceeded in a less effective manner. On the surface of samples immersed in the solution at pH = 9.5, no sulfur was observed based on the results of the XPS. This confirms the previous observation that sulfur is not removed from water at higher pH values.

The lower concentrations of zinc on the iron samples after the second batch tests as compared to the first tests results (see Table 2) may indicate that this element is released to demineralised water to a small extent. In the opinion of the authors, however, the decrease in the zinc concentration after the second test for the initial pH of 7 was too big. Most likely, the analysis after the first and the second tests did not include the same locations.

At the same time, while discussing the results of water analysis, it was found that zinc was not released to water after the second batch tests. No indication of zinc in the water may result from the fact that the samples of water were not digested before the measurement in a spectrophotometer. Zinc in the form of other compounds may be transferred to water. In a very a small extent, zinc may also be released into the DW.

HRES spectrums of O1s obtained from the XPS consist of two peaks with similar binding energy for all the analyzed samples. The peaks were as follows: 530.1 ± 0.2 and 531.6 ± 0.3 eV. They correspond to the metal oxides and metal hydroxides, respectively. The peak at 530.1 ± 0.2 eV, which was a result of a metal-oxygen bond, may correspond to many compounds such as the following: γ-Fe_2_O_3_ (529.8 eV), Fe_3_O_4_ (530.0 eV), FeO (529.8 eV), α-FeOOH (530.1 eV), or even to ZnO (530.3 eV), while the peak at 531.6 ± 0.3 eV corresponds to γ-FeOOH (531.4 ± 0.2 eV). In case of the first peak, it was difficult to decide which compound corresponded to the binding energy of 530.1 eV. Taking into account the results from the diffractometer, however, it may be established that it corresponded either to Fe_3_O_4_, in which the iron atoms have been replaced by zinc atoms or pure magnetite. It should be added that in case of the solutions with the initial pH of 7.0 and 9.5, γ-FeOOH was also formed, but in smaller quantities, because the photoelectric lines were weak. In addition, X-ray diffractograms provided no evidence for the presence of lepidocrocite in the crystalline form (Fig. 1b).

Furthermore, in the Fe2p spectra for all the samples, the binding energy amounted to 711.3 ± 0.3 eV. This value confirms the presence of γ-FeOOH on the surface of the samples (Fe2p 711.3 eV—γ-FeOOH).

In case of the iron samples immersed in solutions with the initial pH values of 4.5, 7.0, and 9.5, the Zn2p3/2 emission line in the registered spectra appeared at the binding energy of 1021.5 ± 0.4 eV. This value corresponds to the ZnFe_2_O_4_ compound. The binding energy of this compound amounts to 1021.4 eV. This value has confirmed, according to the data from the XRD tests, the formation of compounds such as ZnFe_2_O_4_.

As provided by Furukawa et al. (2002), Roh et al. (2000), and Rangsivek and Jekel (2005), the secondary minerals which typically form on the surface of ZVI as a result of purification of water contaminated by metals in ionic forms are as follows: γ-FeOOH, Fe_3_O_4_, Fe_2_O_3_, α-FeOOH, CaCO_3_, Fe(OH)_2_, Fe_2_O_3_·0.5H_2_O, FeCO_3_, FeS_2_, Fe_3_S_4_, mackinawite ((FeNi)_1 + *x*_S, where *x* = 0 to 0.11), and green rust ([Fe_1 − *x*_^2+^ Fe_*x*_^3+^(OH)_2_]^*x*+^ [*x*/*n* A^*n*-^ · mH_2_O]^*x*−^, where *x* is the Fe^3+^/Fe_tot_ ratio), which is usually formed under neutral pH conditions. Although according to Wilkin and McNeil (2003), green rust is a primary corrosion product formed on ZVI in sulfate-rich solutions, it has not been observed (by visual inspection) on the surface of the iron sample and there has been no evidence of it in the presented results. This is probably due to the “pure” conditions of purification. As provided by Cornell and Schwertmann (1996), green rust is stable only at low grades of oxide reduction and its oxidation usually leads to the formation of Fe_2_O_3_ or γ-FeOOH. Roh et al. (2000) also reported that green rust is an intermediate stage and is finally transformed into α-FeOOH, γ-FeOOH, Fe_3_O_4_, and Fe_2_O_3_.

The formation of magnetite (in pure form) was investigated by Grosvenor et al. (2004), Kamolpornwijit et al. (2004), and Karabelli et al. (2008). The latter has noted (on the basis of XRD analyses) a slow development of iron oxides, primarily in the forms of Fe_3_O_4_ and γ-Fe_2_O_3_. Kishimoto et al. (2011) reported that the iron hydroxide formed in earlier stages was finally oxidized and transformed into iron oxides.

In conclusion, based on the results of the XPS and XRD, the products formed on the surface of ZVI as a result of zinc removal from water were the following: (1) magnetite, in which zinc replaces iron creating Zn_x_Fe_3-x_O_4_—in acidic, neutral, and alkaline conditions and (2) lepidocrocite, mainly in acidic conditions. After the second batch tests, i.e., after inserting the samples into demineralised water, the same compounds remained on the ZVI.

Ferrous and ferric ions were formed as a result of the reactions 1, 2, and 3, while Zn^2+^_(*x*)_Fe^2+^_(1 − *x*)_Fe^3+^_2_O_4_ was formed in accordance with Eq. 4:

4 $$ \left(1-x\right){\mathrm{Fe}}^{2+} + 2{\mathrm{Fe}}^{3+} + x{\mathrm{Zn}}^{2+} + 2{\mathrm{H}}_2\mathrm{O} + {\mathrm{O}}_2{{\to \mathrm{Z}\mathrm{n}}^{2+}}_{(x)}{{\mathrm{Fe}}^{2+}}_{\left(1-x\right)}{{\mathrm{Fe}}^{3+}}_2{\mathrm{O}}_4 + 4{\mathrm{H}}^{+} $$

where *x* ≤ 1.

### Specific Surface of Iron Samples

The values of specific surface areas obtained by means of multi-point BET method for the iron samples immersed in the zinc solutions with the pH values of 4.5, 7.0, and 9.5 (after the first batch tests) were 0.2704, 0.3324, and 0.2741 m^2^/g, respectively, while these same parameters for the samples immersed in DW (after the second batch tests) equalled to 0.2482, 0.3521, and 0.2908 m^2^/g. In case of the iron samples before the batch tests, the parameter amounted to 0.0302 m^2^/g, which means that large amounts of chemical compounds have formed on the surface of the iron samples after the tests, especially at the initial pH = 7.0. It may be stated that after inserting the samples into DW, the surface area did not change. Based on these results, either the substances were not released into the water, or new compounds have been created on the surface of the samples.

### pH_pzc_

**Fig. 3:**
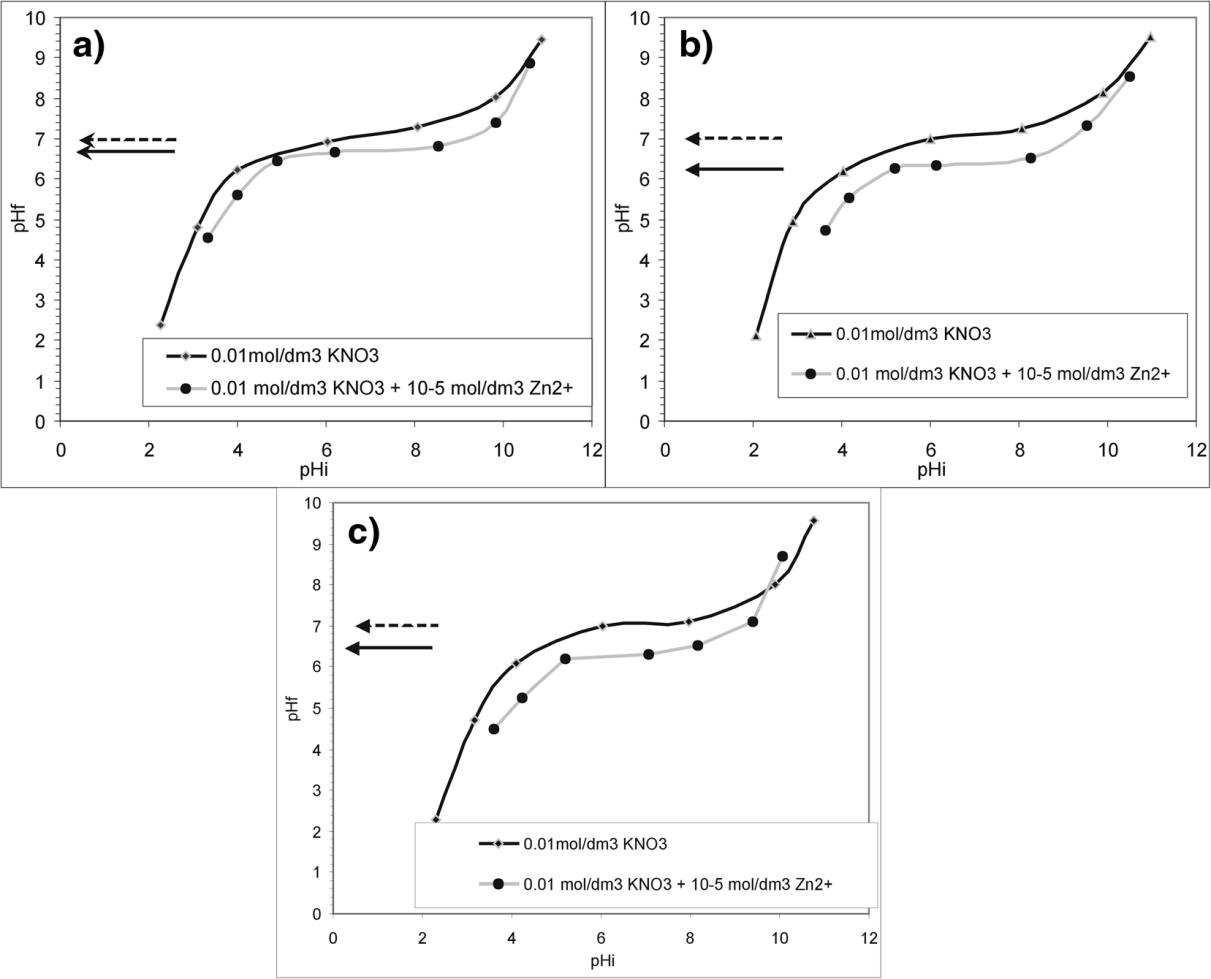
The final value of pH (pH_f_) vs. the initial value of pH (pH_i_) for the iron samples immersed (separately) in two solutions: 0.01 M KNO_3_ and 0.01 M KNO_3_ + 10^−5^M Zn(II). Three samples have been used in each solution: **a** the one that was shaken in a solution with the initial pH = 4.5, **b** the one that was shaken in a solution with the initial pH = 7.0, and **c** the one that was shaken in a solution with the initial pH = 9.5

Figure 3 presents the results of the tests conducted for the determination of pH_pzc_. The pH at the point of zero charge, determined by batch equilibrium method for the three iron samples (that have been used in the zinc solution at the initial pH = 4.5, 7.0, and 9.5) which were shaken in 0.01 M KNO_3_ solutions, amounted to 7 (pH_f_ level at which a common plateau is reached, see the dashed arrows in Fig. 3). This leads to the conclusion that pH_pzc_ is independent from the initial pH of zinc solutions.

In the presence of zinc ions in solutions, pH_pzc_ decreases to the value of ca. 6.7, 6.3, and 6.4 (pH_f_ level at which a common plateau is reached, see the solid arrows in Fig. 3), respectively, for initial pH values of the solutions amounting to 4.5, 7.0, and 9.5. The decrease in pH_pzc_ is due to the specific adsorption of counter ions. The values of point of zero charge for iron oxides (hydroxide) are as follows: 7.8 for lepidocrocite γ-FeOOH, from 3.8 to 8.2 for magnetite, from 6.1 to 7.5 for maghemite γ-Fe_2_O_3,_ from 5.5 to 9.3 for hematite α-Fe_2_O_3,_ from 6.2 to 9.6 for goethite α-FeOOH, and from 6.0 to 6.5 for iron(II) hydroxide (Kosmulski 2011).

Based on the above, it may be stated that the sorption of Zn(II) on the surface of samples is not possible for the solution with the initial pH of 4.5, in case of which the final pH was 5.27 (see Table 1), while it may occur in case of the solutions with the initial pH of 7.0 and 9.5, for which the final pH was 6.31 and 6.85, respectively (see Table 1). The shells coating the iron samples following the tests exhibited nearly equal the amounts of negative and positive charges, as the pH of the solutions (6.31 and 6.85) was close to the pzc of the compounds which have formed on the surface of the iron samples. Since both the charges exist in the shells coating the samples at this pH, the sorption of Zn(II), as well as the negatively charged ions which were present in the solution, may occur at the surface of the samples.

In the research described in the papers by Kishimoto et al. (2011) and Rangsivek and Jekel (2005), the zinc removal process was also enhanced at higher pH values.

## Conclusions

ZVI has the capacity to remove zinc from water in a wide range of pH values. The concentrations of zinc in water decreased much faster in higher pH levels of the solutions. At the same time, zinc was not released to demineralised water from the precipitates located on the ZVI (or was released to a small extent), as DW is not a reducing agent. The results of the XPS indicated, however, that some amount of zinc can be released into demineralised water. The specific surface of iron samples, in turn, did not change after inserting the samples into DW. In this case, no conclusion can be drawn. It can only be stated that zinc may be released into the DW to a very limited extent—if at all.

The products formed on the surface of the ZVI as a result of zinc removal from water were dependent on the initial pH values of the solutions. Under acidic conditions γ-FeO(OH) and Zn_x_Fe_3 − *x*_O_4_ (where *x* ≤ 1) were the main precipitates, while under alkaline conditions, the iron surface was covered with Zn_*x*_Fe_3 − *x*_O_4_ and to a lesser extent with γ-FeO(OH). For lower initial values of pH, the presence of sulfates on the surface of the samples has also been identified. Similar observations to the ones described above were made for the second batch tests, in which the ZVI samples were submerged in demineralised water. To summarize, Zn_*x*_Fe_3 − *x*_O_4_ was the main compound generated as a result of the removal of zinc in ionic form from water in a wide range of pH values.

The adsorption of Zn(II) on the surface of precipitates which have formed on the ZVI may occur as an additional process in neutral and high pH of solutions, since the pH was approximately 6.3–6.4 at point of zero charge for shells coating zero-valent iron. The final pH of these solutions reached the values of 6.31 and 6.85.
